# MSC/MSC-EV therapy for chronic wounds: microenvironmental reprogramming, product design, and clinical translation

**DOI:** 10.3389/fbioe.2026.1960442

**Published:** 2026-10-19

**Authors:** Maojing Xiong, Yutao Xiong, Wanqing Xie, Lu Yang, Bo Yang

**Affiliations:** 1 State Key Laboratory of Oral Diseases & National Center for Stomatology & National Clinical Research Center for Oral Diseases & Department of Post-Anesthesia Care Unit, West China Hospital of Stomatology, Sichuan University, Chengdu, Sichuan, China; 2 State Key Laboratory of Oral Diseases & National Center for Stomatology & National Clinical Research Center for Oral Diseases & Engineering Research Center of Oral Translational Medicine, Ministry of Education & National Engineering Laboratory for Oral Regenerative Medicine & Department of Maxillofacial Plastic, Aesthetic and Trauma Surgery, West China Hospital of Stomatology, Sichuan University, Chengdu, Sichuan, China; 3 State Key Laboratory of Oral Diseases & National Center for Stomatology & National Clinical Research Center for Oral Diseases & Department of Dental Emergency and General Dentistry, West China Hospital of Stomatology, Sichuan University, Chengdu, Sichuan, China

**Keywords:** biomaterials, chronic wounds, clinical translation, extracellular vesicles, mesenchymal stromal cells, microenvironmental reprogramming

## Abstract

Chronic wounds are sustained not by a simple delay in the normal repair sequence, but by a hostile tissue microenvironment—persistent ischemia, non-resolving inflammation, proteolytic matrix degradation, oxidative stress, microbial burden, and senescence collectively form a self-reinforcing network that limits the effectiveness of conventional dressings and single-target interventions. Mesenchymal stromal cells (MSCs) and MSC-derived extracellular vesicles (MSC-EVs) are increasingly viewed not as replacement cells, but as transient, adaptive modulators of this pathological niche; their therapeutic effects are mediated largely through paracrine and vesicle-based signaling, which can recalibrate macrophage and T-cell responses, support angiogenesis, restore extracellular matrix homeostasis, protect resident keratinocytes and fibroblasts, and attenuate oxidative and infectious stress. This review reframes MSC-based therapy for chronic wounds as a product-design problem, synthesizing current evidence from wound biology, MSC and MSC-EV mechanisms, biomaterial-assisted delivery, source selection, potency testing, and early clinical studies, and argues that successful translation will depend on indication-matched products that couple defined biological potency with engineered delivery systems, clinically meaningful endpoints, patient stratification, and regulatory-ready manufacturing controls.

## Graphical Abstract

**Figure:**
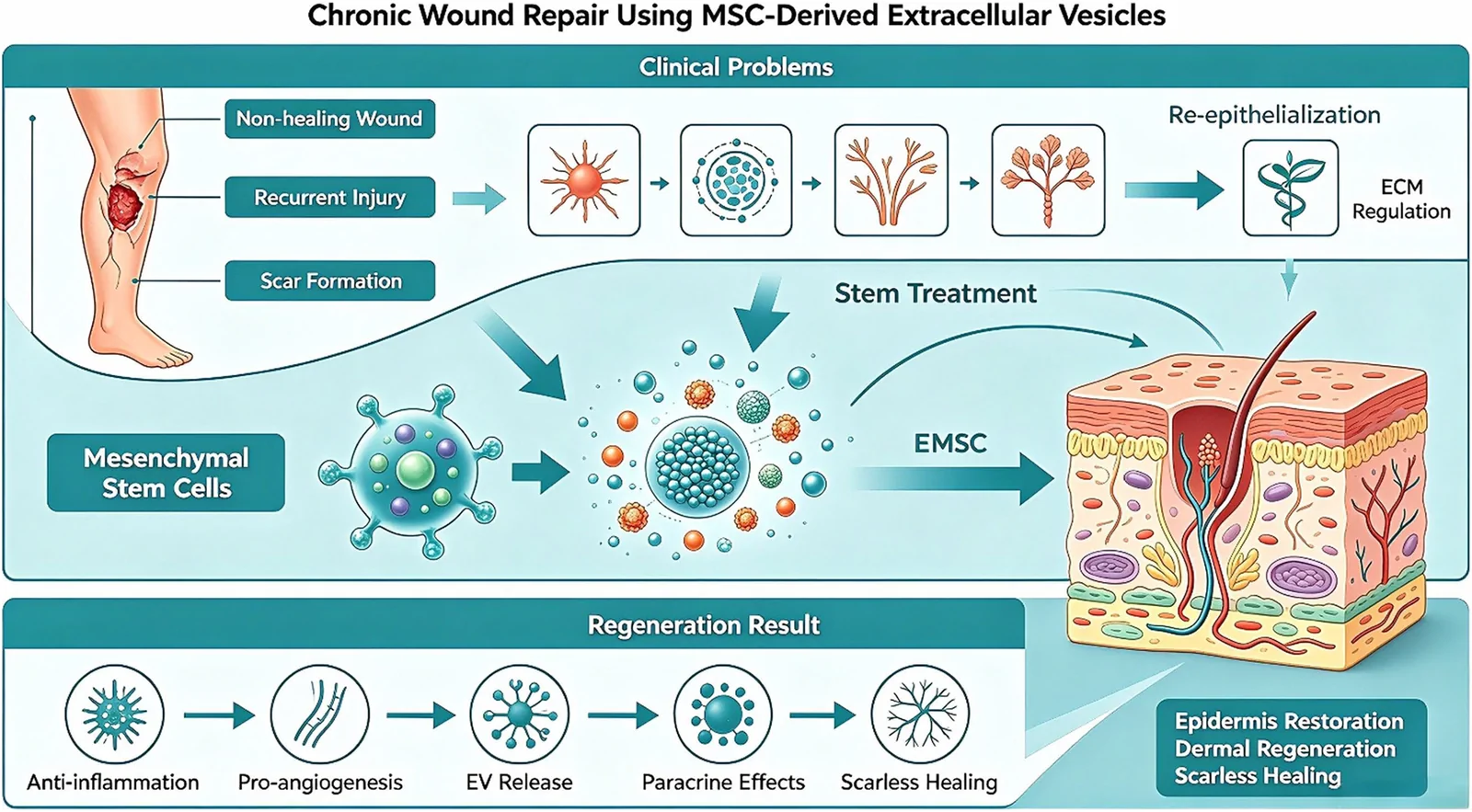

## 1 Introduction

Chronic wounds, including diabetic foot ulcers, venous leg ulcers, pressure injuries, and arterial insufficiency ulcers, are clinically defined by their failure to proceed through orderly tissue repair after 4–8 weeks of standard care (Pastar et al., 2023; Sengul et al., 2025). This definition is useful for diagnosis but misleading for therapy, because it implies a simple delay in a normal sequence. In reality, chronic wounds are trapped in a self-sustaining pathological state where persistent hypoxia, persistent inflammation, excessive protease activity, oxidative stress, microbial colonization, and cellular dysfunction mutually reinforce one another (Atkin et al., 2019; Anders et al., 2019). Optimized standard care remains the foundation of chronic wound management—including debridement, offloading for diabetic foot ulcers (DFUs), compression for Venous leg ulcers (VLUs), pressure redistribution for pressure injuries, and revascularization where feasible for ischemic wounds. Yet a substantial proportion of wounds fail to heal despite guideline-concordant care, because the self-sustaining pathological microenvironment may not be fully reset by addressing individual contributors alone (Schilrreff and Alexiev, 2022; McDermott et al., 2023).

This biological reality has driven interest in mesenchymal stromal cells (MSCs) and MSC-derived extracellular vesicles (MSC-EVs). Early enthusiasm rested on the assumption that MSCs repair tissues by long-term engraftment and differentiation into skin cells or endothelial lineages. That assumption has largely been abandoned (Yan et al., 2025; Renesme et al., 2025). A large body of contemporary evidence supports a more restrained and more actionable view: MSCs act mainly as transient medicinal signaling cells or stromal modulators (Somoza et al., 2016). Their therapeutic effects—and those of their EVs—are mediated through paracrine factors, vesicle-delivered cargo (miRNAs, proteins, lipids), mitochondrial transfer, and short-lived cell-cell interactions (Gorgun et al., 2021; Zhou et al., 2023). In chronic wounds, where the hostile niche rapidly clears transplanted cells, it has been proposed that durable engraftment may not be required for therapeutic benefit, and that a sufficient, well-orchestrated burst of paracrine signals may be sufficient to shift the wound microenvironment toward resolution. However, the relationship between MSC persistence, viability, and therapeutic function remains complex and incompletely understood, and the minimal duration and viability threshold for meaningful clinical effect have not been established.

However, despite hundreds of preclinical studies and a growing number of early-phase clinical trials, no MSC or MSC-EV product has yet received regulatory approval for chronic wounds as a primary indication. The field faces a familiar translational bottleneck: biological plausibility far exceeds clinical proof. We hypothesize that this gap may be attributable in significant part to product-level design challenges—insufficient definition of source, manufacturing, formulation, delivery, potency, and patient selection—rather than to a fundamental lack of MSC biological activity. This hypothesis remains to be rigorously tested in adequately powered, product-specific clinical trials, and biological variability, patient heterogeneity, and trial design limitations likely also contribute. Most studies still ask “do MSCs work?” rather than “which MSC product, for which wound phenotype, delivered by which system, measured by which potency assay, and tested against which endpoint?”.

This review therefore reframes MSC/MSC-EV therapy for chronic wounds as a product-design and translational problem anchored in three linked core questions. First, which pathological microenvironmental barriers must be reprogrammed for a given wound phenotype and clinical indication? Second, which therapeutic format—viable cells, extracellular vesicles, secretome, or biomaterial-assisted combination products—can deliver the required biological activity at the wound bed with sufficient duration, stability, and safety? Third, which clinical trial design and endpoints can demonstrate patient-relevant benefit rather than mere biological plausibility? Guided by this framework, the review is structured sequentially from the chronic wound microenvironment and therapeutic mechanisms, to product engineering, clinical evidence, and a proposed translational roadmap.

This review draws on a structured search of PubMed/MEDLINE, Embase, Web of Science, Scopus, and the Cochrane Library (January 2010–August 2026), combining three search blocks—mesenchymal stem/stromal cell terms, extracellular vesicle/secretome terms, and chronic wound/ulcer terms—with Boolean operators. Only peer-reviewed publications were included. Original research (*in vitro*, animal, human), systematic reviews, meta-analyses, and clinical trial reports were included; conference abstracts, editorials, duplicates, and non-cutaneous wound studies were excluded. Reference lists were hand-searched for additional studies, and key journals and citation alerts were monitored through August 2026 to identify recently published work relevant to the revision. As a narrative review, no formal risk-of-bias or quality assessment tool was applied; instead, clinical evidence was weighted by study design (RCTs > prospective controlled > single-arm prospective > retrospective case series), and evidence from preclinical models was interpreted with explicit caveats regarding translatability.

## 2 Chronic wound microenvironment as the therapeutic target

Acute wound healing progresses through controlled, overlapping phases of haemostasis, inflammation, proliferation, and remodelling (Ellis et al., 2018; Urciuolo et al., 2022). In chronic wounds, this sequence is not simply delayed but derailed. The tissue becomes locked in a self-reinforcing pathological state where four interconnected barriers—ischaemia, unresolved inflammation, proteolytic matrix degradation, and cellular dysfunction—mutually amplify one another (Gallant-Behm et al., 2018; Rodrigues et al., 2019; Wang K. et al., 2020; Fan et al., 2021) (Table 1). A therapy that addresses only one of these barriers is unlikely to restore repair. This section therefore defines each barrier not as a biological curiosity, but as a design constraint for MSC/MSC-EV products.

**TABLE 1:** Comparative overview of key biological features between normal acute healing and chronic wound.

Dimension	Acute healing	Chronic pathology
Ischemia	Transient ischemia, reperfusion is achievable	Persistent ischemia, bioenergetic failure
Inflammation	Self-limiting, resolves in a timely manner	Inflammation is unresolved, persistent pro-inflammatory macrophage activation
Matrix Remodeling	Balanced MMPs/TIMPs, orderly repair and remodeling	Imbalanced MMPs/TIMPs, excessive matrix degradation, structural disorganization
Cell Function	Active proliferation and migration of fibroblasts and keratinocytes	Cellular senescence, proliferation arrest, impaired migratory function

### 2.1 Ischaemia and impaired angiogenesis

Persistent ischaemia, driven by macrovascular occlusion, microvascular rarefaction, and endothelial progenitor cell dysfunction, limits oxygen and nutrient delivery. Hypoxia-inducible factor (HIF)-1α signalling is often dysregulated, reducing expression of vascular endothelial growth factor (VEGF), stromal cell-derived factor 1 (SDF-1), and other pro-angiogenic factors (Comşa et al., 2017; Lupo et al., 2023). The consequence is not merely slow healing but failure to mount a coordinated angiogenic response. For MSC/MSC-EV therapy, long-term engraftment is generally not observed and may not be required; a transient, paracrine angiogenic burst may be sufficient. Restoring perfusion—or enabling tissue survival under sustained hypoxia—becomes the design priority.

### 2.2 Non-resolving inflammation

Chronic wounds are not characterised by an absence of inflammation but by its poor resolution. Damage-associated molecular patterns (DAMPs), microbial products, and necrotic debris sustain nuclear factor kappa-light-chain-enhancer of activated B cells (NF-κB)-driven cytokine production (tumor necrosis factor alpha (TNF-α), interleukin one beta (IL-1β), interleukin 6 (IL-6)) (Gao et al., 2023; Luo et al., 2024). Macrophages remain polarised toward pro-inflammatory (M1-like) states, neutrophil persistence leads to neutrophil extracellular trap cell death (NETosis) and protease release, and adaptive responses often favour T helper 1/T helper 17 (Th1/Th17) over regulatory T-cell activity (Galli et al., 2011; Knopf et al., 2019). This inflammatory milieu directly damages tissue and blocks the transition to proliferation. A product must therefore reprogramme, not simply suppress, immune cells-shifting macrophages toward repair-associated phenotypes and limiting neutrophil-mediated injury.

### 2.3 Proteolytic matrix degradation

Elevated and mismatched matrix metalloproteinases (MMP-2, MMP-8, MMP-9) degrade collagen, fibronectin, growth factors, and integrin-binding structures, while their endogenous inhibitors (tissue inhibitors of metalloproteinases (TIMPs)) are relatively deficient (Cabral-Pacheco et al., 2020; Fang and Lan, 2023). The result is a disorganised extracellular matrix (ECM) that cannot support cell migration or vessel stabilisation. Restoring matrix integrity requires not only inhibiting excess proteases but also re-establishing tissue inhibitor balance and reducing the inflammatory drivers of MMP production. MSC-derived TIMP-1/TIMP-2 and anti-inflammatory signals are mechanistically relevant, but potency assays must confirm matrix-protective activity.

### 2.4 Cellular senescence, oxidative stress, and microbial burden

Fibroblasts and keratinocytes exposed to the chronic wound niche undergo senescence, apoptosis, or migration defects. Oxidative stress (reactive oxygen and nitrogen species) activates proteases and inactivates endogenous inhibitors, while microbial biofilms amplify inflammation and blunt antimicrobial clearance (Resnik et al., 2020; Liu et al., 2022; Wei et al., 2022). An effective product must protect resident cells from oxidative injury, restore their migratory/proliferative capacity, and support host-directed antimicrobial activity. MSC-EVs carrying nuclear factor erythroid 2-related factor 2 (NRF2)-regulatory microRNAs (miRNAs) or antioxidant enzymes are promising, but this cytoprotective function must be measured directly—not inferred from cell-free assays.

### 2.5 The network, not the node

The critical insight for product design is that these barriers are coupled. Ischaemia worsens inflammation; inflammation drives MMP expression; MMPs degrade matrix, which further impairs cell migration and vessel maturation; senescent cells secrete pro-inflammatory and pro-protease factors. Consequently, a therapy that potently blocks a single node (e.g., an MMP inhibitor or an anti-TNF antibody) has repeatedly failed in chronic wounds; network redundancy and adaptive rewiring have been proposed as one contributing explanation (Figure 1). The next section examines how MSCs and MSC-EVs reprogramme each of these barriers, and where the evidence is strong versus still preliminary.

**FIGURE 1:**
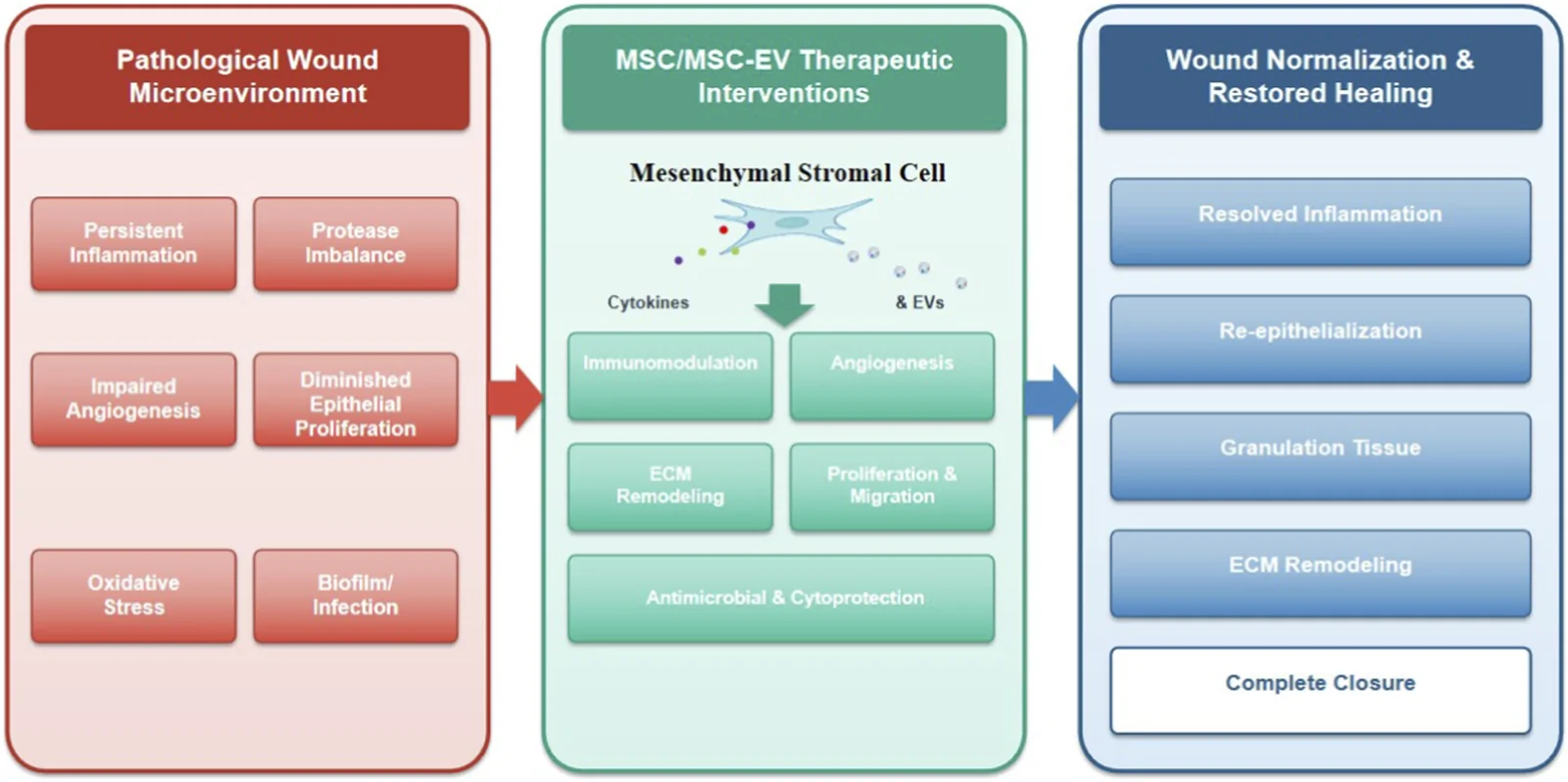
Therapeutic logic of MSC and MSC-EV therapy in chronic wounds: pathological barriers are targeted by MSC/MSC-EV interventions, leading to wound normalization and restored healing.

## 3 MSCs and MSC-EVs as microenvironmental reprogramming agents

### 3.1 From stem-cell replacement to stromal signaling

The historical assumption that MSCs repair tissues by engrafting and differentiating into keratinocytes, endothelial cells, or fibroblasts has largely been abandoned. A large body of evidence now supports a different view: MSCs act mainly as transient, adaptive modulators of the tissue microenvironment. Their therapeutic effects—and those of MSC-derived extracellular vesicles (MSC-EVs)—are mediated through paracrine factors, vesicle-delivered cargo (miRNAs, proteins, lipids), mitochondrial transfer, and short-lived cell-cell interactions (Hu et al., 2018; Brown et al., 2019; Mohammadalipour et al., 2020). In chronic wounds, where the hostile niche rapidly clears transplanted cells, it has been proposed that durable engraftment may not be required, and that a sufficient burst of coordinated paracrine signals may redirect endogenous repair. Nevertheless, the relationship between cell persistence, viability, and functional outcome remains an active area of investigation, and the conditions under which transient versus more sustained delivery is optimal have not been defined.

This framework also clarifies why MSC-EVs and secretome-based products are increasingly important. EVs carry miRNAs, proteins, lipids, and metabolites that can reproduce several MSC functions without introducing viable cells. They may offer off-the-shelf storage, lower embolic and proliferative risks, and easier integration with hydrogels or dressings, although EV manufacturing, potency assays, cargo heterogeneity, and dose metrics remain major barriers (Brennan et al., 2020; Bray et al., 2022; Ma et al., 2023).

### 3.2 Reprogramming the major wound barriers

Table 2 summarises the key MSC/MSC-EV activities for each barrier, along with an evidence-base classification indicating the model systems in which each mechanism has been verified. The takeaway is not that MSCs “do everything”, but that multiple mechanisms have been validated in chronic wound (predominantly diabetic) animal models; however, direct human chronic-wound mechanistic studies and reproducible links to clinical potency remain lacking.

**TABLE 2:** Key activities of MSCs/MSC-EVs against chronic wound pathological barriers and assessment of evidence strength.

Wound barrier	MSC/MSC-EV activity	Therapeutic implication	Direct wound evidence	*In vitro*/Extrapolated	Key limitations
Ischemia and immature angiogenesis	VEGF, bFGF, Ang-1, SDF-1/CXCR4 signalling, pro-angiogenic EV cargo (miR-126, miR-210, miR-100-5p), mitochondrial transfer to endothelial cells	Restores perfusion, supports endothelial survival, vessel maturation, and nutrient delivery (Zhu et al., 2018a; Park et al., 2018; Zhu et al., 2018b; Han et al., 2019; Nakamura et al., 2020; Lee et al., 2021; Vieira et al., 2022; Wang et al., 2022; Su et al., 2025; Huang et al., 2024 )	Multiple STZ-diabetic rodent studies confirming increased CD31^+^ vessel density and VEGF expression	*In vitro*: HUVEC tube formation and migration	Rodent healing by contraction differs from human re-epithelialization; vessel durability and functional perfusion not established in humans; large-animal evidence limited; no human mechanistic data
Non-resolving inflammation	PGE2, TGF-β, IL-10, IDO, HLA-G5, PD-L1, EV miRNAs dampening TLR/NF-κB; dynamic macrophage state transition	Shifts macrophage polarization, supports Treg activity, limits Th1/Th17 and neutrophil-mediated injury (Ortiz-Virumbrales et al., 2020; Wang et al., 2020b; Negi and Griffin, 2020; Aleahmad et al., 2021; Patrick and Annamalai, 2022; Skibber et al., 2022; Zhou and Bai, 2022; Piekarska et al., 2022; Feng et al., 2023; Wang et al., 2022; Yin and shen 2024; Li et al., 2025b; Bai et al., 2025; Teng et al., 2022 )	Multiple STZ-diabetic rodent studies confirming CD206^+^ M2 increase and iNOS/TNF-α decrease	*In vitro*: BMDM polarization assays	Macrophage phenotyping not standardized across studies; timing and durability of state transition unknown; human wound tissue cytokine/macrophage profiling absent in clinical trials; rodent inflammation differs from human chronic inflammation
Proteolytic ECM breakdown	TIMP-1/TIMP-2 secretion, MMP3/TIMP1 ratio modulation, reduced inflammatory MMP induction, fibroblast regulation, balanced collagen I/III deposition	Preserves matrix scaffold, supports granulation tissue and keratinocyte migration (Clarke et al., 2015; Matsui et al., 2017; Hu et al., 2019; Paganelli et al., 2020; Alexandrushkina et al., 2020; Li et al., 2021; Wang et al., 2022; Bailey et al., 2022; Shi et al., 2017 )	Diabetic rodent studies showing increased collagen deposition and modulated MMP/TIMP balance	*In vitro*: fibroblast collagen synthesis Partial non-wound extrapolation: some original MMP/TIMP data from renal fibrosis/cancer models	Predominantly rodent models; human chronic wound ECM differs in composition and stiffness; scar quality and long-term remodeling not assessed; some MMP/TIMP data extrapolated from non-wound models
Resident cell dysfunction and senescence	HGF, IGF-1, EGF-related factors, mitochondrial transfer, NRF2/HO-1 cytoprotection, KEAP1/NRF2 axis modulation	Improves keratinocyte/fibroblast migration, proliferation, and stress resistance (Wang et al., 2020c; Navaratnarajah et al., 2022; Yu et al., 2023; Lee et al., 2023; Liu et al., 2023a; Zhang, 2024; Huang et al., 2024; Yuan et al., 2025; Wang et al., 2025 )	STZ-diabetic rodent studies showing Nrf2 activation and improved re-epithelialization	*In vitro*: high-glucose keratinocyte/fibroblast protection Partial non-wound extrapolation: some senescence data from spinal cord/corneal models	Senescence measurement not standardized; *in vitro* findings may not reflect aged, comorbid human cells; mitochondrial transfer demonstrated mainly *in vitro*; some senescence data extrapolated from non-wound models
Oxidative and microbial stress	Antioxidant enzymes (SOD2, HO-1), NRF2 regulation, antimicrobial peptides, macrophage/neutrophil modulation, ROS-scavenging hydrogel combinations	Reduces secondary tissue injury and supports host-directed antimicrobial defense (Chow et al., 2020; Denu and Hematti, 2021; Xiao et al., 2021; Silva-Carvalho et al., 2022; Wang et al., 2022; Kazemi et al., 2022; Sun et al., 2022; Mozaffari et al., 2025; Zhang et al., 2022a; Wang X et al., 2025; Han et al., 2026 )	Infected diabetic rodent models (*S. aureus*, *E. coli*, MRSA) showing bacterial clearance and ROS reduction	*In vitro*: antioxidant and antibacterial assays	Antimicrobial effect largely indirect via immunomodulation; biofilm models underrepresented; clinical antimicrobial efficacy not separately assessed; no human mechanistic data

### 3.3 The pleiotropy paradox: why “doing many things” is both strength and liability

The fact that MSCs and MSC-EVs can simultaneously influence inflammation, angiogenesis, matrix stability, and cell survival is biologically attractive. In a coupled system like the chronic wound, a proposed explanation for the limited success of single-target interventions (e.g., anti-TNF, MMP inhibitors) is network redundancy and adaptive rewiring, whereby inhibition of one pathway is compensated by upregulation of parallel signaling. This remains a conceptual framework rather than a definitively established mechanism, though it is consistent with the pleiotropic nature of wound repair biology.

However, from a translational perspective, pleiotropy becomes a major liability unless the intended mechanism of action (MoA) is defined and measurable. A product labelled as “MSC therapy” may vary wildly in its dominant activity depending on donor, tissue source, culture conditions, passage number, priming, cryopreservation, and delivery carrier. Without a MoA-linked potency assay, regulators cannot determine whether a bad batch failed because it lacked angiogenic signals, immunomodulatory signals, or both.

Furthermore, MSC-EVs add another layer of complexity: cargo is sensitive to production method, storage, and purification; particle-to-function ratios are inconsistent across laboratories; and most EV studies still do not report active dose in functional units (e.g., angiogenic units or immunomodulatory units) rather than protein or particle count.

The biological rationale for MSC/MSC-EV therapy in chronic wounds is strong but remains insufficiently standardised. Translating this rationale into a reproducible therapy requires moving beyond mechanistic inventories toward engineered delivery, measurable potency, and indication-matched clinical validation.

## 4 Product design: source, format, delivery, and potency

### 4.1 Source selection should be indication-matched

No MSC source is universally optimal. Bone marrow-derived MSCs (BM-MSCs) have the longest historical record and strong stromal repair rationale, but harvest is invasive and donor age or diabetes can reduce function. Adipose-derived MSCs (AD-MSCs) are abundant and attractive for autologous use, with pro-angiogenic and anti-fibrotic secretomes, yet obesity and metabolic disease can impair potency. Umbilical cord-derived MSCs (UC-MSCs) offer allogeneic scalability and strong proliferative capacity; while they have relatively favorable immunological properties due to low MHC-II and co-stimulatory molecule expression, they should not be considered universally immune-privileged, particularly with repeat dosing or manufacturing-associated phenotype changes. Dental-derived MSCs are biologically interesting, particularly for immunomodulatory and pro-angiogenic secretomes, but remain early for chronic wound clinical translation (Huang et al., 2009; Ganier et al., 2018; Mastrolia et al., 2019; Koh et al., 2019; Yan et al., 2021; Preda et al., 2021; Zhang et al., 2021; Malhotra et al., 2022).

A product-design view therefore asks which wound type is being treated, and must incorporate the standard-of-care foundation for each indication. Ischemic diabetic wounds may require angiogenic and metabolic support, with products designed to integrate with offloading, infection control, and vascular assessment; venous leg ulcers may require inflammation and matrix control, with formats compatible with compression therapy and wound bed preparation; pressure injuries may require protection of hypoxic tissue and anti-fibrotic remodeling, working alongside pressure redistribution and nutritional support; and CLTI products are relevant only when revascularization is not feasible. MSC/MSC-EV therapy should be positioned as an adjunct to optimized standard care rather than a replacement. The delivery format, dosing schedule, and clinical trial design should reflect these background care requirements—for example, a hydrogel or dressing format may be more compatible with daily offloading and compression routines than an injectable suspension requiring repeated clinic visits. Ignoring this standard-care context risks designing products that cannot be integrated into real-world wound care pathways.

Beyond source selection, MSC product heterogeneity is driven by manufacturing and handling variables that can substantially alter cell phenotype, secretome composition, and therapeutic efficacy. Single-cell transcriptomic studies have revealed that functionally distinct MSC subpopulations—such as Csf1+ AD-MSCs—can be the key determinant of treatment outcomes, underscoring that bulk population measurements mask critical functional heterogeneity (Hou et al., 2025). Culture conditions—including seeding density, medium formulation, and passage number—drive phenotypic and functional divergence (Fernández-Santos et al., 2022; Calcat-i-Cervera et al., 2023); cryopreservation and post-thaw handling can acutely reduce viability and immunomodulatory function, with partial recovery only after a 24-h acclimation period (Antebi et al., 2019; Moll et al., 2014); and dose, route, and carrier format determine retention, survival, and exposure at the wound bed (Caplan et al., 2019; Eylert et al., 2021). In recognition of these sources of variability, ISCT and a 2025 Delphi-driven consensus have defined standardized product-definition and clinical reporting frameworks, specifying that MSC therapeutics should report: (1) tissue source and donor characteristics; (2) isolation and expansion methods; (3) immunophenotype per ISCT minimal criteria (CD73/CD90/CD105 ≥ 95%, hematopoietic markers ≤2%) (Dominici et al., 2006); (4) viability at release and post-thaw recovery; (5) fresh versus cryopreserved state and storage conditions; (6) dose—including absolute cell number and, where applicable, weight-normalized dose; (7) administration route and carrier/vehicle; and (8) functional potency assays linked to the intended mechanism of action (Renesme et al., 2025). However, our analysis of the chronic wound MSC trials summarized in Table 4 reveals that many of these consensus variables—particularly donor characteristics, isolation/expansion details, full immunophenotyping, viability at release, post-thaw recovery, and functional potency assays—are underreported or absent. This underreporting prevents objective comparison of product differences across studies and is itself a major barrier to identifying which product attributes drive clinical efficacy.

### 4.2 Cell, EV, or combination product

Viable MSC products retain adaptive sensing capacity: inflammatory or hypoxic licensing can amplify immunomodulatory and angiogenic signals. However, viable cells are harder to standardize, may lose function after cryopreservation or injection stress, and often persist only transiently after administration (Yin et al., 2019; Evtushenko et al., 2020; Abbaszadeh et al., 2020; Zhou et al., 2021; Gelibter et al., 2022; Brito and Trentin, 2025). MSC-EVs are more modular and may be easier to combine with wound dressings, hydrogels, microneedles, or electrospun fibers, supporting the broader transition toward cell-free derivatives in wound healing and scar management (Akbari et al., 2026), but EV products need robust identity, purity, cargo, dose, and potency metrics. The relative advantages, principal risks, and translational implications of each product format are summarised in Table 3.

**TABLE 3:** MSC products, MSC-EVs, and combination therapies for chronic wounds: a comparative overview of advantages, challenges, and future directions.

Product format	Design advantage	Main risk	Translational implication
Injectable cell suspension	Simple preparation; local or intramuscular delivery	Rapid washout, apoptosis, uneven distribution	Short signaling window; best when repeated dosing or strong vascular target is planned
Cell-seeded hydrogel/scaffold	Retention, mechanical protection, moist wound interface	Complex CMC; carrier-cell interaction must be defined	Useful for DFU/VLU where sustained local exposure is needed
MSC-EV hydrogel/dressing	Cell-free; extended shelf-life and long-term stability (no cold-chain requirement for cell viability); off-the-shelf availability; compatible with topical care	EV dose unit, cargo stability, release kinetics, and potency remain unresolved	Attractive for outpatient wound-care products
Engineered MSC or EV	Enhanced homing, survival, angiogenic or immunomodulatory cargo	Higher regulatory and safety burden	Best reserved for indications with clear mechanistic deficit and high unmet need
Smart responsive biomaterial	Can release cargo in response to pH, ROS, enzymes, or infection cues	Design complexity and validation burden	Could align therapy intensity with wound-state dynamics

Combination products may be the most realistic path for difficult wounds. Biomaterial carriers can protect cells or EVs, retain them at the wound bed, provide moisture and mechanical support, and respond to local cues such as pH, proteases, or reactive oxygen species (Moon et al., 2019; Wang R. et al., 2019; Yang et al., 2020; Meamar et al., 2021; Zuo et al., 2023). In this setting the carrier is not a passive vehicle but part of the therapeutic product.

Aligning with the MISEV 2023 guidelines (Welsh et al., 2024), several methodological considerations are critical for MSC-EV products. First, the generic term “extracellular vesicle (EV)” should be used rather than “exosome” unless the biogenesis pathway has been definitively demonstrated, which is rarely the case in wound-healing studies. Second, MSC-EVs must be distinguished from broader conditioned medium or secretome products: EVs represent a defined vesicular subset that requires separation from non-vesicular components including protein aggregates, lipoproteins, and residual media factors. Isolation and purification methods—including differential ultracentrifugation, tangential flow filtration, and size-exclusion chromatography—each yield different purity profiles and carry distinct contamination risks. Characterization should include particle size distribution (nanoparticle tracking analysis), morphology (transmission electron microscopy or cryo-EM), tetraspanin markers (CD9, CD63, CD81), and absence of non-EV contaminants. Dose normalization remains a major challenge: particle number or total protein concentration alone cannot establish biological potency, and functional units (e.g., angiogenic or immunomodulatory activity) are needed. Storage conditions also affect EV integrity, with freeze-thaw cycles and prolonged storage potentially altering cargo and function. Finally, source-cell culture conditions and preconditioning—including hypoxia, inflammatory cytokine licensing, and serum deprivation—profoundly influence EV cargo composition and therapeutic activity, and must be reported and controlled for reproducibility.

EV preconditioning through controlled culture conditions is a powerful product-design variable that profoundly impacts cargo composition and potency. Hypoxic preconditioning (1%–5% O_2_) enhances EV production and enriches angiogenic cargo through HIF-1α-mediated metabolic reprogramming. Hypoxic AD-MSC-derived EVs show altered size distribution, enriched wound-healing-related miRNAs, and functionally increased angiogenic and wound-healing potential (Wu et al., 2025). Similarly, EVs from hypoxia-activated human amniotic membrane MSCs display altered proteomic profiles with improved regenerative functionality (Kholgh et al., 2026). Preconditioning parameters—oxygen concentration, duration, serum level—must be standardized and documented as part of product definition, as they directly determine potency and batch consistency.

Beyond methodological standardization, the scale-up of MSC-EV manufacturing from bench to clinical scale presents distinct bioengineering challenges that directly affect product quality and translational feasibility. Upstream, the transition from traditional 2D planar culture to 3D production systems—including hollow fiber bioreactors and microcarrier-based stirred-tank reactors—can increase EV yield by 4- to 140-fold while enabling continuous, closed-system perfusion over weeks (Jakl et al., 2023; Wiest and Zubair, 2025). However, 3D culture conditions alter cell density, nutrient gradients, oxygen tension, and shear stress, all of which can modify EV cargo composition and functional potency; hollow-fiber-produced EVs, for example, may exhibit enhanced immunomodulatory activity compared with 2D-derived EVs, but the underlying mechanisms and reproducibility require systematic characterization. Downstream, the shift from differential ultracentrifugation—effective at laboratory scale but difficult to scale and prone to operator variability—to closed-system tangential flow filtration (TFF) combined with size-exclusion chromatography (SEC) enables processing of 2–6 L batches with up to 36-fold particle concentration while preserving EV integrity and biological activity (Zhou et al., 2026; Ulpiano et al., 2025). Yet TFF membrane cutoff, crossflow rate, and diafiltration volume can selectively retain or lose specific EV subpopulations and protein contaminants, directly affecting purity and particle-to-protein ratios. GMP-compliant manufacturing further requires xenogeneic-free or chemically defined media, single-use closed consumables, donor screening and cell banking, in-process monitoring of cell viability and metabolite levels, and comprehensive release characterization including particle concentration, size distribution, marker expression, purity, sterility, endotoxin, and functional potency. The central challenge across all scale-up stages is batch-to-batch consistency: small variations in seeding density, passage number, perfusion rate, or harvest timing can propagate into substantial differences in EV yield, cargo profile, and therapeutic activity, mandating standardized process parameters, real-time quality analytics, and robust comparability protocols before clinical deployment.

### 4.3 Delivery engineering: from simple injection to smart responsive systems

The delivery system determines whether the therapeutic reaches the wound bed in sufficient concentration for a sufficient duration. Five main platforms are available, each with distinct advantages and unresolved engineering gaps.

Injectable suspension–Simple and clinically familiar, but suffers from rapid washout (hours to a day), uneven distribution, and mechanical stress on cells (Wang X. et al., 2019; Nakao et al., 2023). It is best used when repeated dosing is feasible or when systemic or intramuscular targeting of ischaemic muscle is intended (e.g., critical limb ischaemia). However, there is currently no standard for injection volume, cell density, or vehicle composition (saline, plasma, or buffer).

Hydrogel and scaffold encapsulation–Improves retention and protects cells/EVs from proteases and shear stress. Natural polymers (collagen, alginate, gelatin) offer biocompatibility; synthetic polymers (polyethylene glycol [PEG], poly (lactic-co-glycolic acid) [PLGA]) allow tuning of degradation and release (Zhang et al., 2020; Lee et al., 2020; Song et al., 2023; Ning et al., 2025). Yet most preclinical studies measure retention only at early time points (days) and do not correlate with healing outcomes. The *in vivo* half-life of encapsulated cells or EVs is rarely measured directly, and degradation products may cause inflammation. Decellularized tissue scaffolds represent a distinct category that preserves native extracellular matrix architecture, biochemical composition, and biomechanical properties beyond what synthetic or single-component natural polymers can achieve. Derived from tissues including articular cartilage, amniotic membrane, dermis, and adipose tissue, these scaffolds serve as biological substrates for cell-free therapies by providing structural support, retaining endogenous growth factors and matrix-bound cytokines, and guiding cell infiltration and neotissue formation. Recent preclinical evidence demonstrates that decellularized bovine articular cartilage scaffolds significantly accelerate diabetic wound closure (100% vs. 75% at day 17) with enhanced epidermal regeneration and granulation tissue formation (Akbari et al., 2025), while three-dimensionally decellularized human amniotic membrane scaffolds exhibit favorable porous architecture, collagen fiber organization, and biological properties suitable for wound dressing applications (Heidari et al., 2025). Limitations include batch-to-batch variability, potential residual immunogenicity, and challenges in sterilization and standardization.

Microneedle arrays–Enable minimally invasive, sustained delivery into the dermis with high patient acceptability, making them attractive for outpatient wound care (Chi et al., 2020; Tekko et al., 2020; Lyu et al., 2023). Current limitations include low loading capacity (typically <10^5^ cells or microgram amounts of EVs) and difficulty in achieving multi-dose or responsive release. Notably, microneedle designs for EV delivery remain at an early prototype stage, with no clinical wound data available.

Wound dressings (fibrous, foam, film) – Easiest to integrate into existing wound care workflows. Electrospun nanofibre dressings can incorporate EVs or secretome and provide moisture balance and physical protection (Li et al., 2022; Sadeghi-Avalshahr et al., 2025). However, loading efficiency and release kinetics are often poorly controlled, and most dressings do not respond to wound state changes. A major challenge is reproducible manufacturing of EV-loaded dressings with defined release profiles.

Smart responsive systems–Designed to release cargo in response to pH (alkaline in infected wounds), reactive oxygen species (elevated in chronic inflammation), or bacterial enzymes (proteases, lipases). These systems could align therapy intensity with wound dynamics (Chowdhury et al., 2022; Yang et al., 2022). Nevertheless, their complexity increases regulatory burden, and few have been tested in large animal chronic wound models. Validation of responsiveness in real-time and demonstration of clinical added value over non-responsive controls are still lacking.

The timing of MSC-EV application relative to the dynamic wound microenvironment is an underappreciated efficacy determinant. Chronic wounds fluctuate between hyper-inflammatory and hyper-proteolytic states, with dynamic changes in pH, MMP activity, and biofilm burden; applying EVs during peak proteolytic phases can degrade cargo and lose potency before reprogramming occurs. Wound bed preparation (TIME framework: Tissue, Infection, Moisture, Edge) may serve as biological priming to reduce protease burden before EV application. Whether formal sequential strategies—debridement/antimicrobials followed by staged EV dosing, or phase-matched anti-inflammatory then pro-angiogenic formulations—are necessary remains open. Stimuli-responsive hydrogels (pH-, ROS-, or protease-triggered) offer an engineering solution by protecting EVs during hostile phases and releasing them when the microenvironment becomes permissive (Harries et al., 2016; Tang et al., 2025; Jia et al., 2026; Li S. et al., 2025).

In summary, both the choice of delivery system and the timing of application should be driven by the wound’s physical and biological environment, not by convenience. A product intended for a highly exudative, infected wound needs different release, retention, and timing properties than one for a dry, ischaemic ulcer. Delivery format and application schedule should be co-designed: protease-protective or stimuli-responsive systems may enable application during hostile microenvironmental phases, while wound bed preparation and staged dosing may improve efficacy when the microenvironment is highly dynamic.

### 4.4 Potency assays must reflect the wound mechanism

Minimal identity markers—including ISCT surface marker criteria and trilineage differentiation potential—establish product identity but do not predict chronic wound efficacy. A regulator-ready MSC or MSC-EV program should distinguish five interconnected levels: (1) identity and characterization (source, phenotype, morphology); (2) critical quality attributes (CQAs) linked to the intended mechanism of action; (3) functional potency assay(s) that measure biological activity relevant to the indication; (4) release criteria defined as statistically justified acceptance ranges; and (5) clinical relevance, meaning the assay output should be correlated—where possible—with therapeutic outcomes (FDA, 2011; FDA, 2023). This hierarchy moves beyond the assumption that identity markers alone can assure therapeutic activity.

Potency assurance is broader than a single lot-release assay. The FDA potency-assurance framework emphasizes a multifaceted, risk-based strategy encompassing manufacturing process design, raw material control, in-process testing, and final lot-release assays, each tied to the intended therapeutic effect (FDA, 2023). For chronic wound products, this means controlling upstream variables—donor selection, culture conditions, passage number, and cryopreservation—that are known to alter MSC secretome and immunomodulatory function, rather than relying solely on end-product testing. Process consistency and in-process controls are particularly important for pleiotropic products where a single release assay may not capture all clinically relevant activities.

For pleiotropic MSC or EV products, a matrix or orthogonal assay strategy is more appropriate than implying that one assay can represent overall wound-healing activity (Galipeau et al., 2016; Niekamp et al., 2026). A wound-relevant potency matrix could include: macrophage state transition or T-cell suppression for immunomodulation; endothelial tube formation and VEGF/HGF secretion for angiogenesis; MMP inhibition or collagen deposition assays for matrix stabilization; keratinocyte scratch migration for epithelial repair; and oxidative-stress rescue assays for cytoprotection (Lee et al., 2016; Liu Y. et al., 2023). Recent work has validated multi-factor potency matrices—incorporating IDO1, M-CSF, EV secretion, and chemokine profiles—that consistently predict *in vitro* functionality across manufacturing lots. The choice of assays should be product-specific and justified by the proposed mechanism of action, with orthogonal methods providing complementary rather than redundant information.

For EV products, potency should not rely only on particle count or total protein. Cargo signatures, vesicle purity, uptake by target cells, release kinetics from the carrier, and biological response in wound-relevant functional assays are needed. Ideally, these assays should be calibrated against clinical endpoints such as complete wound closure, time to closure, recurrence, infection control, pain, and amputation-free survival. The MISEV 2023 guidelines further emphasize that particle number or protein concentration alone cannot establish biological potency (Welsh et al., 2024).

Beyond potency testing, regulatory standardization remains a critical translational barrier for EV products. The MISEV 2023 consensus guidelines provide a foundational framework for EV characterization, identity, purity, and quality control that aligns with regulatory expectations across jurisdictions (Welsh et al., 2024). However, EV products face divergent regulatory classification: the US FDA regulates most EV therapeutics as biologics or drugs requiring IND/BLA pathways and GMP manufacturing; In the EU, ATMP classification is determined on a product-specific basis following scientific recommendation by the EMA Committee for Advanced Therapies (CAT); medicinal-product status alone does not establish ATMP classification, and MSC-derived EV products require case-by-case assessment depending on manufacturing process and intended mechanism of action; and the Iranian IFDA has established specific categorization frameworks for extracellular particles with dedicated guidance on wound-healing applications (Nazari et al., 2026). These classification differences directly impact manufacturing requirements, release specifications, stability study design, and clinical trial endpoints. The absence of harmonized global EV-specific regulatory standards—compounded by cargo heterogeneity and the lack of universally accepted dose metrics—remains a significant obstacle to consistent clinical development and cross-jurisdictional comparability.

## 5 Evidence hierarchy: mechanism-by-mechanism translation from bench to bedside

Before examining the clinical evidence, it is essential to critically acknowledge the limitations of preclinical wound models, as these directly shape the interpretability of translational findings. Rodent skin possesses the panniculus carnosus—a thin subcutaneous muscle layer absent from human skin except as a vestigial structure in the neck (platysma) and scrotum (dartos)—which enables wounds to heal primarily through rapid contraction rather than through re-epithelialization and granulation tissue formation, the dominant mechanisms in human chronic wounds (Zomer and Trentin, 2018; Ojeh et al., 2025). This fundamental difference means that preclinical efficacy signals may partly reflect enhanced contractile closure rather than the epithelial regeneration and matrix deposition that matter clinically. Splinted excisional wound models, which use silicone rings to physically prevent contraction, partially shift healing toward re-epithelialization and improve human relevance, but they introduce their own artifacts—including foreign-body reactions, local mechanical stress, suture-point micro-injury, and frequent device failure due to chewing—and remain acute rather than truly chronic models (Zhang et al., 2026; Fischer et al., 2023). Porcine models offer closer anatomical and physiological similarity to human skin, healing primarily through re-epithelialization with minimal contraction, but they are limited by high cost, poor genetic tractability, specialized housing requirements, and an inherently robust healing capacity that makes true chronicity difficult to achieve (Nunan et al., 2014; Ghetu et al., 2025). For MSC/MSC-EV therapy specifically, these model limitations carry direct translational implications: products that accelerate contraction-dependent closure in rodents may not reproduce the same benefit in human wounds where re-epithelialization and durable granulation tissue are the relevant endpoints, and the acute, metabolically normal environment of most preclinical models fails to capture the sustained hyperglycemia, ischemia, biofilm, and senescence that characterize human chronic wounds. This caveat should be borne in mind when interpreting the mechanism-by-mechanism evidence chain that follows.

Human studies of MSC-based therapy for chronic wounds have established acceptable short-to medium-term safety and provided encouraging efficacy signals across diabetic foot ulcers, venous leg ulcers, and pressure injuries. However, the evidence base remains markedly heterogeneous in wound etiology, cell source, autologous versus allogeneic design, route, dose, carrier, background standard care, and endpoint definitions, and most trials are early-phase, small, or proof-of-concept. To evaluate this evidence more rigorously, we trace the translational chain mechanism-by-mechanism—from *in vitro* observation through preclinical chronic wound models to clinical observation (Table 4)—revealing where the evidence is coherent and where it breaks down.

**TABLE 4:** Clinical application of MSCs for chronic wounds: a summary of evidence and heterogeneity across indications.

Study (Year)	Indication/Severity	Design/Phase/N	MSC product	Dose/Route/Frequency	Control/SOC	Endpoint/Follow-up	Efficacy finding	Safety & limitations
PACE III (Norgren et al., 2024)	CLTI, Rutherford 5; unsuitable for revascularization	RCT, Phase III, N = 213 (2:1)	PLX-PAD allogeneic placental MSC; expanded	IM; 30 injections on day 0 and day 60 (0.5 mL each); total 300 × 10^6^	Placebo IM + standard care	Major amputation-free survival; 12 months	Null: amputation/death 28.6% vs. 33%, HR 0.93 (0.53–1.63), P = 0.788. Post-hoc: HbA1c<6.5% HR 0.46, P = 0.048	Well-tolerated; no unexpected SAE. Limitations: post-hoc subgroup; single product
Moon et al. (2019)	DFU, Wagner 1–2; ≥4 weeks; 1–25 cm^2^	RCT, N = 59	Allogeneic ASC sheet; expanded	Topical; single; 1 × 10^6^ cells/film	Standard moist dressing	Complete closure; 8/12 weeks	8 weeks: 73% vs. 47% (P = 0.038); 12 weeks: 82% vs. 53%; median closure 28.5 days	No treatment-related SAE; low 2-year recurrence. Limitations: small; single center
Uzun et al. (2021)	DFU, Wagner 1–2	Phase I/2 safety study, N = 20 (10/10)	Allogeneic AD-MSC; expanded	Intralesional; single; 6 × 10^6^ cells	Standard care	Healing rate; 4-year follow-up	9/10 (90%) treatment vs. 8/10 (80%) control; faster healing (31.0 vs. 54.8 days, P = 0.002)	No adverse reactions. Limitations: very small; single center; cost higher
Hashemi et al. (2021)	DFU, chronic diabetic wounds; age 30–60	Case series, N = 5 (no control)	Allogeneic WJ-MSC seeded on acellular amniotic membrane; expanded	Topical; every 3 days for 9 days; 2 × 10^6^ cells on amniotic membrane	Standard wound care	Wound size reduction; 9days +1 month follow-up	Wound size reduced 93.92% at 9 days; significantly decreased healing time (P < 0.002)	No adverse events. Limitations: no control group; very small sample; scaffold confounds cell-specific effect
Falanga et al. (2022)	VLU, refractory to standard/advanced therapies	RCT, double-blind, 3-arm pilot, N = 11	Autologous BM-MSC; expanded	Topical fibrin spray; single; 1 × 10^6^ cells/cm^2^ wound bed	Saline/fibrin spray placebo	Healing rate; 24 weeks	MSC group accelerated linear healing ∼10-fold vs. saline/fibrin controls (P < 0.01); controls no meaningful closure by 24 weeks	Safe. Limitations: very small pilot; 3-arm design; autologous delay
Kerstan et al. (2022)	Treatment-refractory chronic VLU (failed 3months standard care)	Phase I/IIa, single-arm, N = 31	Allogeneic ABCB5^+^ dermal MSC; expanded	Topical; 1–2 applications; 1 × 10^6^ cells/cm^2^	None (single-arm) + standard compression	Wound size reduction; 12 weeks	21 (70%) responders (≥30% reduction); median area reduction 76% (FAS); 6 (20%) fully closed at 12 weeks	Safe, well-tolerated; only 3/83 TEAEs product-related. Limitations: single-arm no control; highly refractory population; COVID early termination
Gupta et al. (2023) [Stempeucel®]	CLTI due to atherosclerotic PAD; Rutherford III-5/III-6 (ABI≤0.6); ischemic ulcers; no-option	Single-arm, Phase III label extension, N = 24 (22 completed)	Allogeneic pooled BM-MSC (Stempeucel®); expanded	IM calf + perilesional; single; 2 × 10^6^ cells/kg	None (single-arm) + standard care	Rest pain, ulcer healing, ABI, walking distance, AFS; 12 months	Rest pain 8.0→1.1 (P < 0.0001); 82.1% ulcers completely healed at 12 months; ABI improved (P = 0.0001); walking distance +828% (P = 0.0068)	No serious AEs. Limitations: single-arm no control; small sample; label extension design; industry-sponsored
Mei et al. (2025) meta-analysis	DFU (pooled RCTs)	Systematic review and meta-analysis; 6 RCTs (pooled)	Mixed (BM-MSC, ASC, UC-MSC)	Mixed routes/doses	Standard care	Complete closure; amputation; recurrence; SAE	RR = 1.63 (1.23–2.16) for closure; no improvement in amputation, recurrence, or SAE	No increased SAE. Limitations: heterogeneity; small total N; publication bias; GRADE low–moderate

Immunomodulation and inflammation resolution. *In vitro*, MSCs and MSC-EVs consistently shift macrophage populations toward pro-resolving states, reducing TNF-α and IL-1β while increasing IL-10 and TGF-β, rather than enforcing a simplistic M1-to-M2 binary switch (Wang et al., 2022; Yin and shen 2024; Huang et al., 2024). This immunomodulatory capacity translates directly in diabetic rodent wound models, where MSC treatment reduces inflammatory cell infiltration, accelerates the transition from inflammatory to proliferative phases, and improves closure rates (Bai et al., 2025; Li W. et al., 2025; Bailey et al., 2022). In patients, the clinical correlate is less directly measured: trials generally do not report serial wound cytokine profiles or macrophage phenotyping, and improved closure is attributed to combined mechanisms rather than isolated immunomodulatory effects. The clinical evidence for inflammation resolution is therefore inferred from healing outcomes rather than directly demonstrated, representing a gap in mechanism-to-clinic translation.

Angiogenesis and perfusion restoration. *In vitro*, MSCs and MSC-EVs promote endothelial proliferation, migration, and tube formation through VEGF, HGF, and miRNA-mediated pathways (Bai et al., 2025; Li W. et al., 2025; Bailey et al., 2022). In diabetic animal models, this translates to increased capillary density, improved blood perfusion, and enhanced granulation tissue formation (Shi et al., 2017; Su et al., 2025). In the clinic, angiogenic benefit is most directly relevant to ischemic wounds. The PACE Phase III trial in critical limb ischemia—designed explicitly to test whether the allogeneic placental MSC product PLX-PAD could improve perfusion and limb salvage—failed to meet its primary endpoint of major amputation-free survival at 12 months (HR 0.93, P = 0.788), with similar revascularization rates between groups (Norgren et al., 2024). This negative result is specific to the PLX-PAD product, intramuscular injection route, and Rutherford 5 CLTI population tested. A post-hoc subgroup benefit in patients with HbA1c<6.5% (HR 0.46, P = 0.048) is hypothesis-generating. Thus, for angiogenesis—the mechanism with the strongest preclinical rationale in ischemic wounds—the highest-level clinical evidence (from the tested PLX-PAD product in Rutherford 5 CLTI) is neutral, illustrating a clear breakdown in the translational chain.

Proliferation and re-epithelialization. *In vitro*, MSCs and MSC-EVs enhance keratinocyte and fibroblast proliferation and migration via PI3K/Akt and ERK signaling (Yuan et al., 2025; Zhang Y. et al., 2022). In diabetic rodent models, this corresponds to accelerated re-epithelialization, thicker granulation tissue, and improved collagen organization (Shi et al., 2017). In clinical studies, enhanced re-epithelialization is the most consistently observed benefit: randomized controlled trials report higher complete closure rates in DFUs (Moon et al., 2019: 73% vs. 47% at 8 weeks; Uzun et al., 2021: 90% vs. 80% healing), and VLU pilot studies show accelerated wound area reduction (Falanga et al., 2022: ∼10-fold faster linear healing; Kerstan et al., 2022: 76% median area reduction at 12 weeks). A case series of WJ-MSC-seeded amniotic membrane for DFU reported 93.92% area reduction at 9 days (Hashemi et al., 2021), while a single-arm Phase III study of allogeneic BM-MSC for ischemic CLTI ulcers reported 82.1% complete ulcer healing at 12 months (Gupta et al., 2023). No adequately powered RCT has been conducted for pressure injuries specifically. A 2025 meta-analysis of six DFU RCTs confirmed a pooled RR of 1.63 for complete closure, with benefit more apparent in smaller ulcers (Mei et al., 2025). However, the same meta-analysis found no significant improvement in amputation rates, recurrence, or serious adverse events, and most trials follow patients for only 8–24 weeks—too short to establish whether accelerated closure translates to durable, recurrence-free healing.

The gap at the top: durable clinical benefit. Across all three mechanisms, the evidence forms a coherent chain from *in vitro* biology through preclinical efficacy to early clinical signals and controlled short-term closure. The chain breaks at the highest level—clinically meaningful durable benefit. The only completed randomized, placebo-controlled Phase III trial (PACE, testing PLX-PAD in CLTI) was neutral on its primary endpoint. The single-arm Phase III label-extension study of Stempeucel® in CLTI reported encouraging wound healing rates but lacks a control arm and cannot be considered definitive efficacy evidence. No MSC therapy has demonstrated reduced amputation, sustained closure beyond 6 months, improved quality of life, or mortality benefit in adequately powered, multicenter trials with long-term follow-up. Early-phase studies (Kerstan et al., 2022; Falanga et al., 2022; Uzun et al., 2021) provide encouraging safety and efficacy signals but are limited by small samples, single-arm designs, and heterogeneous product definitions. The field’s central challenge is therefore not whether MSCs can influence wound biology—they consistently do across mechanisms—but whether specific, well-characterized products can convert short-term closure into durable, patient-important outcomes. This translational gap justifies the emphasis on product design, potency assurance, and standardized endpoints in the present review.

## 6 Translational roadmap: from proof-of-concept to proof-of-precision

### 6.1 Safety and *in vivo* fate: route-specific considerations

The safety and *in vivo* fate of administered MSCs are not uniform across delivery routes, and route-specific biology should inform product design and clinical trial planning (Shokoohmand et al., 2026). Rather than treating MSC delivery as a uniform approach, it is important to recognize that topical/local, intramuscular, and intravenous administration have distinct biological and safety profiles—including differences in cell persistence, biodistribution, host immune interactions, and adverse event risks—that should inform product design and clinical trial planning.

For viable MSCs administered systemically by intravenous infusion, the dominant early event is pulmonary sequestration: 50%–80% of infused cells lodge in the lung microvasculature within minutes, with most losing viability within 24–72 h (Caplan et al., 2019; Ferrini et al., 2021). The mechanism underlying this “pulmonary first-pass effect” remains debated. One view attributes it primarily to physical size: culture-expanded MSCs measure 15–30 μm in diameter—substantially larger than lymphocytes (4–12 μm) and exceeding the ∼8 μm caliber of pulmonary capillaries—leading to passive mechanical entrapment (Krueger et al., 2018; Szydlak, 2021). Consistent with this, 3D spheroid culture for 48 h reduces MSC diameter by ∼35% and markedly enhances pulmonary passage and distal organ migration (Ji et al., 2024). An alternative view proposes that pulmonary transit is not merely a size filter but an activation or selection step: MSCs surviving passage through the lung may be mechanically or biochemically primed by the pulmonary microenvironment, and the apoptotic cells retained in the lung can drive macrophage efferocytosis and systemic immunomodulation—suggesting that entrapment itself may be therapeutically functional rather than purely a pharmacokinetic limitation (Zheng et al., 2022). Critically, whether reducing cell size to enhance pulmonary passage translates into improved therapeutic efficacy remains unresolved, and this question has direct implications for manufacturing design. Upon contact with blood, MSCs can also trigger the instant blood-mediated inflammatory reaction (IBMIR), characterized by complement activation (C3a), coagulation cascade activation (thrombin-antithrombin complex, D-dimer), and platelet consumption. A key mediator is tissue factor (TF/CD142), whose expression varies widely by tissue source and increases with passage number; highly procoagulant MSC products have been associated with thromboembolic adverse events (Moll et al., 2022).

Local or intralesional delivery avoids the pulmonary first-pass and systemic coagulation risks of IV administration, but retention is limited by the proteolytic, hypoxic, and inflammatory chronic wound microenvironment. MSCs injected into wound tissue face rapid clearance by host immune cells and enzymatic degradation, with persistence typically measured in days rather than weeks. Local delivery does, however, enable direct interaction with tissue-resident immune cells, fibroblasts, and endothelial cells, and may generate higher local concentrations of paracrine factors compared with systemic infusion. Biomaterial-assisted delivery—hydrogels, scaffolds, films, and dressings—fundamentally alters MSC fate by improving retention, protecting cells from proteases and oxidative stress, and providing a supportive three-dimensional microenvironment. Controlled-release platforms can extend paracrine signaling duration, while adhesion molecule-functionalized scaffolds may improve cell survival and spatial localization. The delivery context thus influences not only persistence but also the mechanism through which therapeutic activity is generated: a biomaterial-embedded MSC may act primarily as a sustained secretion source, whereas an IV-infused MSC may act predominantly through apoptotic efferocytosis and systemic immune education.

Acellular MSC-EV products may reduce certain hemocompatibility concerns associated with viable cell infusion, as they do not contain intact cells that trigger contact-phase coagulation. However, EV hemocompatibility is product- and route-dependent: surface tissue factor (TF/CD142) expression and procoagulant activity have been reported for some MSC-EV preparations, with variability depending on cell source, culture conditions, and isolation method (Chance et al., 2019). EV cargo can also carry soluble TF or procoagulant phospholipids, and systemic administration may still activate complement or platelets in a subset of products. However, EVs present their own pharmacokinetic and safety considerations that remain incompletely characterized. In the wound bed, locally applied free EVs undergo rapid clearance through proteolytic degradation, exudate dispersal, and phagocyte uptake, with bioactive signal limited to hours to days unless retained by hydrogels or scaffolds; systemically, approximately 99% of intravenously administered EVs are eliminated within 24 h by reticuloendothelial uptake in the liver and spleen (Bray et al., 2021). On safety, the pro-tumorigenic potential of MSC-EVs must be critically evaluated rather than assumed absent: their cargo includes VEGF, TGF-β, FGF, and miRNAs with documented both pro- and anti-tumorigenic effects depending on cellular source and context (Gilazieva et al., 2022; Huang et al., 2024). This theoretical risk is particularly relevant in chronic wound populations, who frequently carry risk factors for undiagnosed malignancy—older age, diabetes, and chronic inflammation—and may receive repeated or long-term EV exposure. Short-term clinical trials, typically lasting weeks to months with limited post-treatment surveillance, cannot establish long-term oncogenic safety; systematic long-term follow-up and preclinical tumorigenicity studies are therefore warranted before broad clinical deployment.

### 6.2 Mechanism-guided product development: five central requirements

The next phase of MSC/MSC-EV therapy for chronic wounds should move from empirical application to mechanism-guided product development. As outlined in Table 5, five requirements are central. First, products must be defined by intended biological function, not only by source tissue and surface markers. Second, delivery systems must be chosen according to the wound barrier they are meant to solve: retention, survival, controlled release, protection from proteases, infection-responsive release, or outpatient usability. Third, trials must enroll biologically coherent patient groups rather than combining wounds with very different vascular, metabolic, and inflammatory states.

**TABLE 5:** Key steps and goals in the translational development of mechanism-guided MSC/MSC-EV products for chronic wounds.

Development step	Key variables	Goal
Define wound phenotype	DFU, VLU, pressure injury, CLTI; ischemia, infection, protease burden, senescence	Homogeneous enrollment and mechanistic endpoint selection
Choose therapeutic format	MSC, MSC-EV, secretome, engineered EV, cell-biomaterial combination	Indication-matched product rather than source-driven choice
Build mechanism-based potency	Macrophage, endothelial, keratinocyte, fibroblast, MMP, oxidative-stress assays	Reproducible release criteria linked to intended action
Engineer delivery	Retention, release kinetics, scaffold mechanics, degradation, injectability, dressing compatibility	Therapeutic exposure at the wound bed
Design trial and monitoring	Blinded imaging, complete closure, recurrence, amputation-free survival, biomarker substudies	Evidence strong enough for clinicians, regulators, and payers

Fourth, endpoints must be clinically meaningful and aligned with regulatory definitions. A three-tier framework helps distinguish early biological signals from clinically meaningful outcomes: (1) early biological endpoints include changes in wound fluid cytokines, macrophage phenotype, perfusion, and keratinocyte proliferation; (2) surrogate endpoints include percentage area reduction, which is useful for early-phase screening but insufficient as a primary outcome; and (3) clinically meaningful endpoints include complete closure—defined by FDA guidance as re-epithelialization without drainage or dressing requirement, confirmed at two consecutive visits 2 weeks apart (FDA, 2006)—time to closure, durable closure sustained through at least 3–6 months of follow-up, recurrence, infection, pain, quality of life, and amputation-free survival. Embedded biomarker studies using wound fluid, tissue, imaging, microbiome, and systemic metabolic data could identify responders and connect product potency with clinical outcome.

Fifth, regulatory and manufacturing planning should begin early. Cell and EV products face requirements for donor screening, sterility, identity, purity, potency, stability, lot consistency, cryopreservation, and release testing. Combination products add carrier characterization, release kinetics, degradation behavior, and device-biologic interactions. Autologous, allogeneic, minimally manipulated, substantially manipulated, viable-cell, acellular, and engineered products may follow different regulatory pathways. A translational review should therefore treat regulation as a design constraint rather than an afterthought.

## 7 Conclusion

MSC and MSC-EV therapies are best understood as strategies to reprogram the chronic wound microenvironment. Their promise lies in coordinated modulation of inflammation, angiogenesis, matrix remodeling, epithelial repair, oxidative stress, and host defense. Their limitation is the same biological complexity that makes them attractive: unless source, culture, priming, formulation, delivery, potency, and patient selection are controlled, the therapeutic signal will remain difficult to reproduce.

Future progress should therefore prioritize product-level design. The most convincing next-generation therapies will be indication-matched, mechanism-tested, biomaterial-enabled where appropriate, and evaluated in trials with meaningful endpoints and embedded biomarkers. Under this framework, MSCs and MSC-EVs may become rational components of precision wound care, not because they are universal regenerative agents, but because they can be engineered and tested as microenvironmental reprogramming products.
